# Altered Fronto‐Cingulate Connectivity During Social Competition and Cooperation in Adolescents With Major Depressive Disorder: An fNIRS Hyperscanning Study

**DOI:** 10.1155/da/8894844

**Published:** 2026-10-12

**Authors:** Yangliuqing He, Yiming Wang, Shuo Jiang, Fenrong Liang

**Affiliations:** ^1^ Department of Psychosomatic-Sleep Medicine Zigong Mental Health Center Zigong Sichuan China; ^2^ Clinical Medicine College Guizhou Medical University Guiyang China gmc.edu.cn; ^3^ Department of Psychiatry Affiliated Hospital of Guizhou Medical University Guiyang China gmcah.cn

**Keywords:** adolescent depression, competitive behavior, cooperative behavior, fNIRS, functional connectivity, parent–child interaction, social cognition

## Abstract

**Background:**

Major depressive disorder (MDD) in adolescence is characterized by high prevalence (24.3% in China) and social dysfunction, particularly in competitive and cooperative behaviors. This study used fNIRS hyperscanning to explore cortical connectivity alterations during parent–adolescent interactions in adolescent MDD.

**Methods:**

Thirty‐three MDD adolescents (DD) and 29 healthy controls (HCs) completed competitive/cooperative maze tasks with parents under 52‐channel fNIRS. Wavelet coherence analyzed functional connectivity (FC) in prefrontal/motor regions, which provides undirected connectivity estimates. Group × task effects were tested via GLMM and FDR correction.

**Results:**

(1) Competition: DD exhibited hyperconnectivity in emotion regulation circuits between right opercular inferior frontal gyrus and left triangular inferior frontal gyrus (rIFGoperc and lIFGtriang, *p* = 0.047) but hypoconnectivity in executive networks between left dorsal superior frontal gyrus and left precentral gyrus (lSFGdor and lPreCG, *p* = 0.040). (2) Cooperation: DD showed enhanced motor–cognitive integration between right supplementary motor area and right middle frontal gyrus (rSMA and rMFG, *p* = 0.036) but impaired socioemotional processing between right triangular inferior frontal gyrus and left dorsal superior frontal gyrus (rIFGtriang and lSFGdor, *p* = 0.033). Task modulation: Within DD, cooperation preferentially engaged emotional resonance networks between right inferior orbital frontal gyrus and right medial superior frontal gyrus (rORBinf and rSFGmed, *p* = 0.031) while competition was associated with enhanced coupling in self‐monitoring circuits between left dorsal superior frontal gyrus and right opercular inferior frontal gyrus (lSFGdor and rIFGoperc, *p* = 0.047).

**Conclusions:**

Adolescent MDD involves context‐dependent neural deficits in social cognition, suggesting targets for neuromodulatory interventions.

## 1. Introduction

Major depressive disorder (MDD), characterized by persistent low mood [1], exhibits pronounced prevalence during adolescence—a developmental period marked by heightened sensitivity to environmental stressors that elevate risks of emotional dysregulation and depressive symptoms [2–5]. Globally, up to 34% of adolescents report depressive symptoms [6], with prevalence reaching 24.3% in China [7]. Alarmingly, 50–70% experience recurrence within 5 years of remission, frequently culminating in pervasive social dysfunction and long‐term health burdens [8].

Social dysfunction constitutes a core feature of depression, impairing patients’ capacity to fulfill normative social roles [9, 10]. Critically, these deficits persist beyond symptomatic remission [9] and are exacerbated in adolescents by increasing autonomy and peer‐dependence [11]. Substantial evidence links such impairment to altered social cognition—the ability to interpret and respond to others’ mental states [12, 13]. Within this domain, competition and cooperation represent fundamental modes requiring dynamic monitoring of self/other behaviors and adaptive psychological strategies.

Depressed individuals exhibit avoidance of competitive contexts, correlating positively with symptom severity [14]. The social competition hypothesis posits depression as an unconscious failure strategy, promoting submission to maintain safety and emotional stability [15, 16]—a view corroborated by clinical associations between depressive episodes and competitive stress [17, 18]. Neurobiologically, social competition dysfunction in depressed adolescents is mainly linked to abnormal activation of cortical regions supporting emotion appraisal and cognitive control, including ventromedial prefrontal cortex (vmPFC) and insula [19–23], which are well within the detection depth of fNIRS. Conversely, cooperative social interaction relies heavily on cortical executive circuits [24] such as the dorsolateral prefrontal cortex (dlPFC) and inferior frontal gyrus; atypical hemodynamic activity in these cortical areas is consistently reported in depression and leads to interpersonal coordination deficits [25, 26].

Despite the centrality of competition and cooperation in social cognition, three critical gaps persist: (1) Existing studies predominantly examine these processes in isolation, neglecting their integration in naturalistic social contexts; (2) Neural mechanisms underlying social cognition in adolescent depression remain underexplored; and (3) No research has quantified parent–adolescent brain synchrony during ecologically valid social tasks.

To address these gaps, we employed functional near‐infrared spectroscopy (fNIRS)—a noninvasive, motion‐tolerant neuroimaging tool that monitors cortical hemodynamics to infer neural activation. Using a dual‐task hyperscanning paradigm, we investigated prefrontal‐motor functional connectivity (FC) in medication‐naïve depressed adolescents and their parents during competitive and cooperative interactions. This approach elucidates the circuit‐level mechanisms of social dysfunction, informing targeted interventions. Notably, fNIRS only reliably captures hemodynamic signals from cortical gray matter with limited penetration depth and cannot measure subcortical nuclei such as the amygdala and ventral striatum. Therefore, the present study exclusively focuses on prefrontal, cingulate, and motor cortical circuits to match the imaging modality’s measurement capacity.

## 2. Materials and Methods

### 2.1. Participants

This study recruited 33 adolescents diagnosed with major depressive disorder (DD; 15 males, 18 females, aged 13–18 years) from the Psychiatry Department of the Affiliated Hospital of Guizhou Medical University and 30 healthy control adolescents (HC; 14 males, 16 females, aged 13–18 years) from local Guiyang high schools. One HC participant was excluded in preprocessing due to severe, uncorrectable signal artifacts across all fNIRS channels, leading to a final valid sample of 29 HC adolescents. All participants and their legal guardians provided written informed consent prior to data collection. The study was approved by the Ethics Committee of Guizhou Medical University (Approval No. 2023 [763]) and registered with the China Clinical Trial Registry (ChiTR2400081712). Data collection spanned from May 2023 to January 2024. Given the exploratory nature of this dyadic fNIRS hyperscanning paradigm, no prior effect sizes regarding prefrontal–cingulate synchrony during parent–adolescent competitive and cooperative interactions have been reported in the existing literature. Therefore, no formal a priori sample size calculation was conducted. The final sample size was determined according to clinical recruitment feasibility and benchmarked against comparable sample sizes from previous fNIRS studies focusing on adolescent psychiatric populations.

### 2.2. Inclusion and Exclusion Criteria

#### 2.2.1. DD Group

Inclusion criteria: (1) Adolescents aged 13–18 years, regardless of gender; written informed consent was obtained from both participants and their legal guardians; (2) Diagnosed with depressive disorder meeting the criteria of the Diagnostic and Statistical Manual of Mental Disorders, Fifth edition (DSM‐5) by two psychiatrists with attending physician or higher professional titles; the total score on the 24‐item Hamilton depression rating scale (HAMD‐24) was ≥20; (3) First depressive episode, or no antidepressants, anesthetics, or sedative‐hypnotic medications taken for at least 3 consecutive months prior to enrollment; (4) Normal intellectual function, right‐handed, native Chinese speaker, capable of reading Chinese instructions fluently and completing all experimental tasks cooperatively; (5) The accompanying primary caregiver parent meets the healthy control (HC) parent inclusion criteria listed below.

Exclusion criteria: (1) Comorbid neurological disorders or other psychiatric disorders (including intellectual disability); (2) Severe physical illnesses, such as cardiac, hepatic, renal dysfunction, or endocrine diseases; (3) Uncorrectable visual impairment that cannot be compensated by corrective lenses; (4) Intolerance to the fNIRS cap and scanning procedure.

#### 2.2.2. HC Group

Healthy adolescents matched to the DD group in age, gender, and years of education were recruited as controls.

Inclusion criteria: (1) Adolescents aged 13–18 years, regardless of gender; written informed consent was obtained from both participants and their legal guardians; (2) No lifetime DSM‐5 psychiatric disorder diagnosis; the total HAMD‐24 score was <8; (3) No prior use of antidepressants, anesthetics, or sedative‐hypnotic agents; (4) Normal intellectual function, right‐handed, native Chinese speaker, capable of reading Chinese instructions fluently and completing all experimental tasks cooperatively; (5) The accompanying primary caregiver parent must comply with all inclusion criteria below.

#### 2.2.3. HC Parent Inclusion Criteria

(1) Biological primary caregivers (father or mother) of adolescent participants aged 35–55 years; (2) No lifetime DSM‐5 diagnosis of depressive disorder, bipolar disorder, psychotic disorders, severe anxiety disorders, or intellectual disability confirmed via standardized brief clinical interview; (3) No history of severe neurological disease, traumatic brain injury, or uncontrolled chronic systemic physical illness; (4) No history of long‐term dependence on anesthetics, sedatives, hypnotics, or other psychoactive substances; (5) Normal or corrected‐to‐normal vision, capable of fully understanding task instructions and finishing cooperative and competitive maze tasks with adolescents.

Exclusion criteria: All exclusion criteria were identical to those applied to the DD group.

### 2.3. Clinical Assessment

Demographics (age, gender, and education) were collected via structured questionnaires. Depression severity was assessed using the HAMD‐24 by trained clinicians (inter‐rater reliability: ICC > 0.85).

### 2.4. Study Methodology

#### 2.4.1. Experimental Protocol

The experiment comprised two 5‐min task modules—cooperative and competitive—preceded and separated by 30‐s rest periods (eyes closed) to establish baseline psychological and physiological states and minimize carryover effects (total duration: 11 min). The protocol was structured as follows (Figure 1): Initial rest (30 s)→Task block 1 (5 min, cooperative or competitive, counterbalanced across participants)→Inter‐task rest (30 s)→Task block 2 (5 min, alternate task).

**Figure 1:**
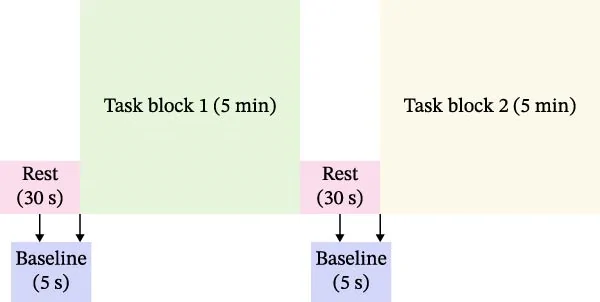
Experimental design diagram.

Adolescents performed tasks with their parents while wearing fNIRS systems, selected for their noninvasive measurement of cortical hemodynamic activity and sensitivity to FC in naturalistic settings. Task order was counterbalanced to control for order effects, and standardized instructions ensured consistent execution.

#### 2.4.2. Task Descriptions

Competitive task (Figure 2): Participants and parents started at the same point in separate, identical mazes and raced to reach the exit first, prioritizing speed and efficiency.

**Figure 2:**
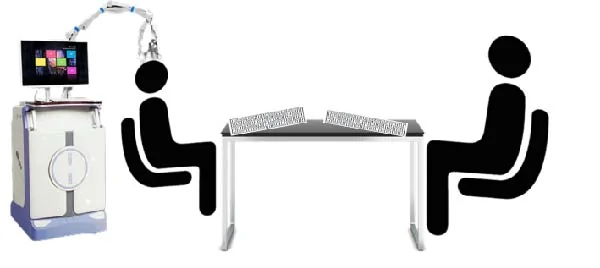
Schematic diagram of the competition task.

Cooperative task (Figure 3): Participants and parents started at opposite maze ends (start and exit points) and collaborated to converge at any midpoint via mutual observation and route adjustment.

**Figure 3:**
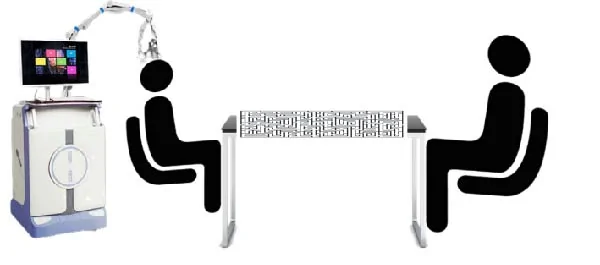
Schematic diagram of the cooperation task.

A 30‐s closed‐eye rest period precedes each task to standardize participants’ psychological and physiological states, with the final 5 s used as a baseline for measuring variables such as heart rate and stress levels. Before each task, participants and their parents are instructed to adopt a competitive mindset for the competitive task or to prioritize cooperative path adjustments based on mutual observation for the cooperative task.

### 2.5. fNIRS Data Acquisition

The fNIRS data were collected using the BS‐3000 system (Wuhan Zilian Hongkang Medical Technology, China), featuring dual‐wavelength laser diodes (670 and 830 nm) optimized for tissue penetration and hemoglobin absorption contrast. Changes in oxygenated (HbO) and deoxygenated hemoglobin (HbR) concentrations were quantified using the modified Beer–Lambert law at a 20‐Hz sampling rate to resolve rapid hemodynamic dynamics. The optode array, consisting of 16 sources and 16 detectors on a commercial fNIRS cap with a 3‐cm source‐detector separation, generated 53 valid channels, each defined as the midpoint of a source‐detector pair. Optodes were positioned per the international 10–20 EEG system, with FPz as the reference and symmetric alignment along the T3–T4 axis. Channels were mapped to Brodmann areas (BA) using standardized anatomical templates for accurate cortical localization [27] (Figure 4).

**Figure 4:**
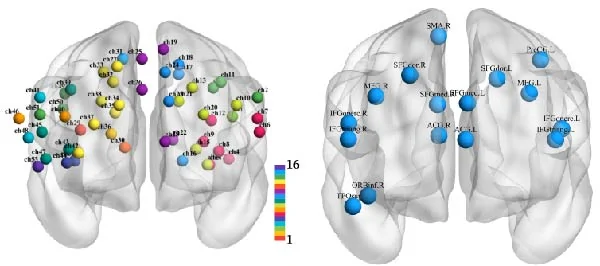
Spatial distribution of fNIRS channels (*n* = 53) on the scalp, aligned with the international 10–20 system and mapped to prefrontal and cingulate Brodmann areas (BA) via standardized anatomical templates.

### 2.6. Statistical Analysis

#### 2.6.1. Data Preparation

The fNIRS data were collected from 33 adolescents with depression (DD group) and 30 HCs group using the Zilian Hongkang BS‐3000 system. One HC participant was excluded due to poor signal quality across all channels (final HC: *n* = 29). Raw signals were converted to optical density using manufacturer‐provided scripts and processed in MATLAB (R2023a) with the Homer2 toolbox [28].

#### 2.6.2. Preprocessing Pipeline

1. Quality control: Channels with signal intensity <0.0001, >10, or mean‐to‐standard deviation ratio <3 were excluded via enPruneChannels (*n* = 2, channels 10 and 40) [29].
2. Artifact correction: Motion artifacts (fluctuations >3× mean or >30× standard deviation within a 1‐s window) were identified using hmrMotionArtifactByChannel and corrected via cubic spline interpolation (hmrMotionCorrectSpline, *p* = 0.99).
3. Signal filtering: A third‐order Butterworth filter (0.01–0.1 Hz) was applied using hmrBandpassFilt to remove physiological noise (e.g., cardiac, respiratory) and low‐frequency drift [30]. Optical density data were converted to oxygenated hemoglobin changes (Δ[HbO]) using the modified Beer–Lambert law with differential pathlength factors of [6, 6] for 690 nm and 830 nm wavelengths.
4. Spatial mapping: About 51 valid channels were parcellated into 16 prefrontal and cingulate regions of interest (ROIs) aligned with the 10–20 system and BA (Figure 4) [31].

#### 2.6.3. FC Analysis

1. Baseline correction and downsampling: Δ[HbO] time series were baseline‐corrected using the 10‐s pre‐task period and downsampled to 1 Hz.
2. Wavelet transform coherence (WTC): Pairwise FC was computed via WTC in the 0.01–0.1 Hz frequency band (task duration: 5 min), generating undirected connectivity matrices per participant and condition.
3. Statistical transformation: Connectivity values were Fisher’s *Z*‐transformed to ensure normality for statistical analysis.

### 2.7. Statistical Analysis

Demographic and clinical differences between groups were assessed using independent *t*‐tests for normally distributed continuous variables, Mann–Whitney *U* tests for non‐normal continuous variables, and chi‐square tests for categorical variables. The Shapiro–Wilk test was used to examine the normality of all datasets.

Given the 2 (Group: DD/HC) × 2 (Task: Cooperative/competitive) factorial design, we adopted generalized linear mixed models (GLMM) for FC analysis. Group, Task, and the Group × Task interaction term were entered as fixed factors, and participant ID was set as a random intercept to account for repeated measurements of two tasks from each adolescent. Fisher’s *Z*‐transformed connectivity values were taken as dependent variables.

If the Group × Task interaction reached statistical significance, post‐hoc pairwise simple effect comparisons were conducted to decompose the interactive pattern. Independent *t*‐tests were further performed to describe group differences under a single task condition, and paired *t*‐tests were used to compare cooperative versus competitive connectivity within the DD group as supplementary analyses.

False discovery rate (FDR) correction was applied to all multiple comparisons to control Type I error, with adjusted *p*  < 0.05 defined as statistically significant. All statistical analyses were implemented in IBM SPSS Statistics 26 and MATLAB R2023a. Normally distributed data are presented as mean ± standard deviation; non‐normal data are reported as median [interquartile range]; categorical variables are summarized as counts (%). Only FDR‐corrected statistical results are reported in the main text.

## 3. Results

### 3.1. Demographic Characteristics of the Study Population

The study included 62 adolescents: 33 with depression (DD group) and 29 HCs group, with one HC participant excluded post‐preprocessing due to poor signal quality. No significant intergroup differences were observed in gender distribution (*χ* ^2^ = 0.002, *p* = 0.961), age (*Z* = −1.522, *p* = 0.128), or years of education (*Z* = −1.408, *p* = 0.159; all *p* > 0.05). In contrast, Hamilton depression rating scale (HAMD) scores differed significantly between groups (*Z* = −6.809, *p* < 0.001; Table 1).

**Table 1:** Demographic and clinical characteristics.

Variable	DD group (*n* = 33)	HCs group (*n* = 29)	*χ* ^2^	*p*‐Value
Gender, *n* (%)	—	—	0.002	0.961
Male	15(45.5%)	13(44.8%)	—	—
Female	18(54.5%)	16(55.2%)	—	—
	—	—	*Z*	*p*‐Value
Age (years), median [IQR]	15 [14, 16]	16 [14, 16]	−1.522	0.128
Education (years), median [IQR]	10 [8, 11]	11 [8.5,11]	−1.408	0.159
HAMD score, median [IQR]	25 [21.5,27]	0 [0,2]	−6.809	＜0.001 ^∗^

*Note:* Data presented as median [IQR] for non‐normally distributed variables.

Abbreviation: HAMD, Hamilton depression rating scale.

^∗^Significant at *p* < 0.05 after FDR correction.

### 3.2. Group Differences in FC During Competitive Tasks

During parent–adolescent competitive tasks, the DD group showed significantly stronger FC (FDR‐corrected *p* < 0.05) in multiple prefrontal and cingulate circuits compared to HCs (Table 2; Figures 5 and 6). Key findings include as follows:

**Figure 5:**
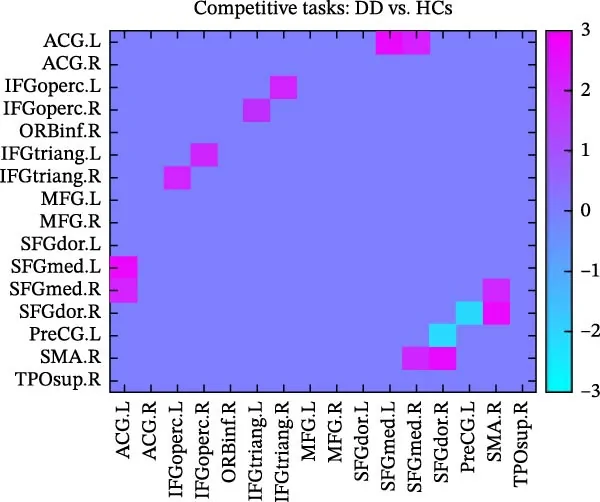
*T*‐value matrix of brain functional connectivity differences between the DD and HCs groups during competitive tasks. (Note: This *T*‐value matrix displays significant results. Purple represents positive *T*‐values, blue represents negative *T*‐values, with color intensity indicating *T*‐value magnitude, as shown in the color bar).

**Figure 6:**
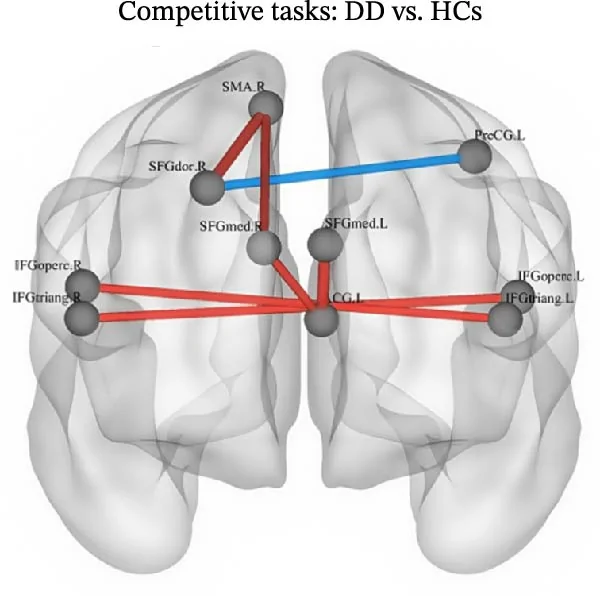
Spherical stick figure of brain functional connectivity differences between the DD and HCs groups during competitive tasks. (Note: This ball‐and‐stick diagram shows significant brain regions (gray spheres) and their connections. Red lines indicate positive *t*‐values, blue lines indicate negative *t*‐values, with thicker lines representing larger *t*‐value magnitudes).

**Table 2:** Between‐group differences in FC during competitive tasks.

Source region	Target region	*t*(60)	*p* (FDR)	Effect direction
IFGoperc.R	IFGtriang.L	2.029	0.047 ^∗^	DD > HC
IFGoperc.L	IFGtriang.R	2.081	0.042 ^∗^	DD > HC
ACG.L	SFGmed.L	2.667	0.010 ^∗^	DD > HC
ACG.L	SFGmed.R	2.146	0.036 ^∗^	DD > HC
SMA.R	SFGmed.R	2.038	0.046 ^∗^	DD > HC
SMA.R	SFGdor.R	2.579	0.012 ^∗^	DD > HC
SFGdor.L	PreCG.L	−2.097	0.040 ^∗^	DD < HC

Abbreviations: ACG, anterior cingulate gyrus; IFG, inferior frontal gyrus; PreCG, precentral gyrus; SFGdor, superior dorsal frontal gyrus; SFGmed, superior medial frontal gyrus; SMA, supplementary motor area.

^∗^ *p*  < 0.05 (FDR, corrected).

Increased connectivity between IFGoperc. R and IFGtriang. L (*t*(60) = 2.029, *p* = 0.047) and between IFGoperc. L and IFGtriang. R (*t*(60) = 2.081, *p* = 0.042).
ACG. L showed enhanced connectivity with SFGmed. L (*t*(60) = 2.667, *p* = 0.010) and SFGmed. R (*t*(60) = 2.146, *p* = 0.036).
SMA. R exhibited stronger connections with SFGmed. R (*t*(60) = 2.038, *p* = 0.046) and SFGdor. R (*t*(60) = 2.579, *p* = 0.012).
Decreased connectivity between SFGdor. L and PreCG. L (*t*(60) = −2.097, *p* = 0.040).

### 3.3. Group Differences in FC During Cooperative Tasks

During parent–adolescent cooperative tasks, the DD group exhibited significantly stronger FC (FDR‐corrected *p* < 0.05) in multiple prefrontal and cingulate circuits compared to HCs (Table 3; Figures 7 and 8). Key findings include as mentioned below:

**Figure 7:**
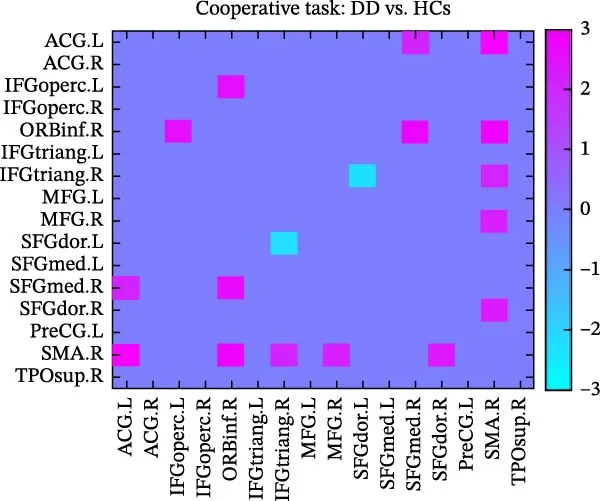
*T*‐value matrix of brain functional connectivity differences between the DD and HCs groups during cooperative tasks.

**Figure 8:**
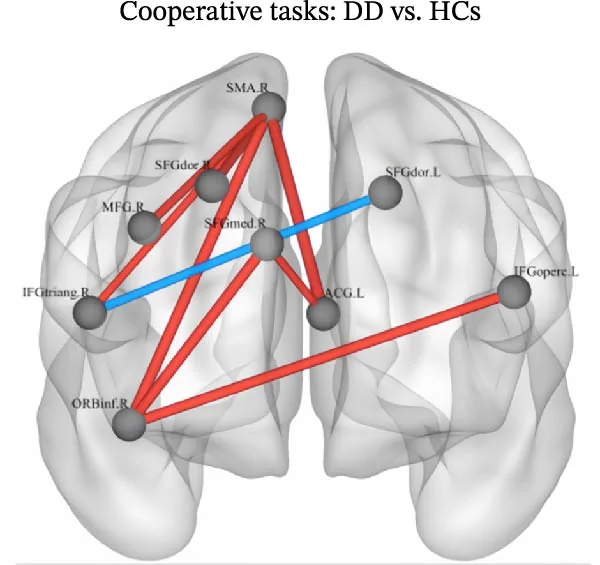
Spherical stick figure of brain functional connectivity differences between the DD and HCs groups during cooperative tasks.

**Table 3:** Between‐group differences in FC during cooperative tasks.

Source region	Target region	*t* (60)	*p* (FDR)	Effect direction
ACG.L	ACG.R	2.031	0.047 ^∗^	DD group > HCs group
ORBinf.R	SFGmed.R	2.616	0.011 ^∗^	DD group > HCs group
SMA.R	ACG.L	2.846	0.006 ^∗^	DD group > HCs group
SMA.R	ORBinf.R	2.748	0.008 ^∗^	DD group > HCs group
SMA.R	IFGtriang.R	2.043	0.046 ^∗^	DD group > HCs group
SMA.R	MFG.R	2.153	0.036 ^∗^	DD group > HCs group
SFGdor.R	SMA.R	2.264	0.027 ^∗^	DD group > HCs group
IFGtriang.R	SFGdor.L	−2.183	0.033 ^∗^	DD group < HCs group

Abbreviations: ACG, anterior cingulate gyrus; IFGtriang, triangular part of inferior frontal gyrus; MFG, middle frontal gyrus; ORBinf, orbital part of inferior frontal gyrus; SFGdor, superior dorsal frontal gyrus.; SFGmed, superior medial frontal gyrus; SMA, supplementary motor area.

^∗^ *p*  < 0.05 (FDR, corrected).

Increased connectivity between IFGoperc.L and IFGorb.R (*t*(60) = 2.528, *p* = 0.014).
Enhanced connectivity between ACG.L and ACG.R (*t*(60) = 2.031, *p* = 0.047).
Increased connectivity between IFGorb.R and SFGmed.R (*t*(60) = 2.616, *p* = 0.011).
SMA.R showed stronger connections with ACG.L (*t*(60) = 2.846, *p* = 0.006); IFGorb.R (*t*(60) = 2.748, *p* = 0.008); IFGtriang.R (*t*(60) = 2.043, *p* = 0.046); MFG.R (*t*(60) = 2.153, *p* = 0.036); and SFGdor.R (*t*(60) = 2.264, *p* = 0.027).
Decreased connectivity between IFGtriang.R and SFGdor.L (*t*(60) = −2.183, *p* = 0.033).

### 3.4. Group × Task Interaction Effects on FC

The above separate analyses of competitive and cooperative tasks demonstrated distinct intergroup connectivity differences under each social context. To further verify whether these group differences vary as a function of task type, we conducted GLMM analysis incorporating the Group × Task interaction term.

GLMM analysis revealed significant Group × Task interaction effects on several prefrontal–cingulate connectivity pairs (all FDR‐corrected *p*  < 0.05). Post‐hoc simple effect tests clarified the task‐specific group divergence:

For connectivity between lSFGdor and lPreCG: The DD group exhibited significantly weaker coupling than HC only during competitive tasks, while no significant intergroup difference was observed under cooperative interaction.

For connectivity between rORBinf and rSFGmed: Hyperconnectivity in the DD group was exclusively detected in cooperative tasks, with comparable connectivity strength between DD and HC during competitive tasks.

For SMA‐related motor–cognitive circuits (SMA–MFG, SMA–SFGdor): DD showed significantly elevated connectivity relative to HC in cooperation, whereas intergroup differences were attenuated in competitive contexts.

These interaction results confirm that abnormal frontocingulate connectivity in adolescent depression is highly dependent on social task context, rather than a uniform trait across cooperative and competitive interpersonal interactions.

### 3.5. Within‐DD Group Task Effects: Cooperative vs. Competitive Tasks

To further characterize the task‐driven connectivity reconfiguration specifically within the depressed group, we performed paired comparisons between cooperative and competitive conditions.

Within the DD group, cooperative tasks elicited stronger connectivity in prefrontal control networks compared to competitive tasks (Table 4; Figures 9 and 10).

**Figure 9:**
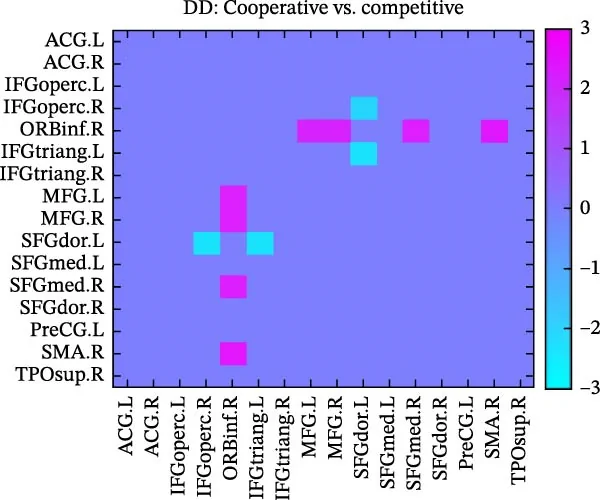
*T*‐value matrix of functional connectivity in DD group during competitive and cooperative tasks.

**Figure 10:**
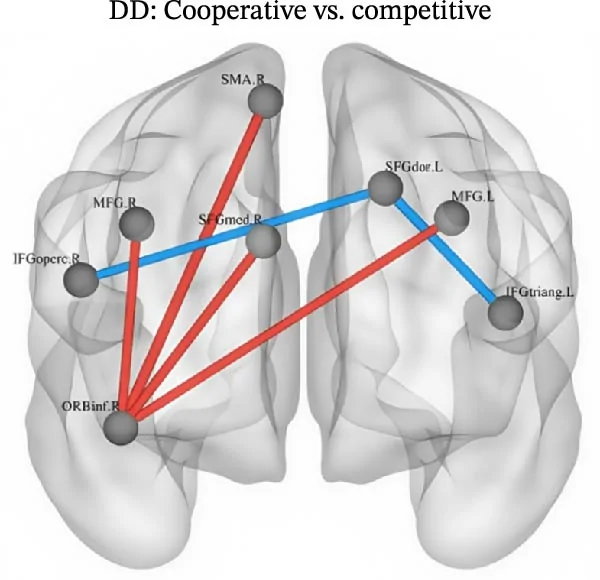
Spherical stick figure showing differences in brain region connectivity in DD group during competitive and cooperative tasks.

**Table 4:** Within‐group differences in FC during competitive vs. cooperative tasks in adolescents with depression.

Source region	Target region	*t*	*p* (FDR)	Effect direction
ORBinf.R	MFG.L	2.133	0.041 ^∗^	Coop > Comp
ORBinf.R	MFG.R	2.162	0.038 ^∗^	Coop > Comp
ORBinf.R	SFGmed.R	2.251	0.031 ^∗^	Coop > Comp
ORBinf.R	SMA.R	2.491	0.018 ^∗^	Coop > Comp
SFGdor.L	IFGoperc.R	−2.066	0.047 ^∗^	Coop < Comp
SFGdor.L	IFGtriang.L	−2.322	0.027 ^∗^	Coop < Comp

Abbreviations: Comp, competitive task; Coop, cooperative task; IFGoperc, opercular part of inferior frontal gyrus; IFGtriang, triangular part of inferior frontal gyrus; MFG, middle frontal gyrus; ORBinf, orbital part of inferior frontal gyrus; SFGdor, superior dorsal frontal gyrus; SFGmed, superior medial frontal gyrus; SMA, supplementary motor area.

^∗^ *p* < 0.05 (FDR, corrected).

Increased connectivity: ORBinf.R with MFG.L (*t* = 2.133, *p* = 0.041); ORBinf.R with MFG.R (*t* = 2.162, *p* = 0.038); ORBinf.R with SFGmed.R (*t* = 2.251, *p* = 0.031); and ORBinf.R with SMA.R (*t* = 2.491, *p* = 0.018).

Decreased connectivity:

SFGdor.L with IFGoperc.R (*t* = −2.066, *p* = 0.047); SFGdor.L with IFGtriang.L (*t* = −2.322, *p* = 0.027).

## 4. Discussion

This study utilized fNIRS hyperscanning to investigate brain FC in adolescents with depression (DD) during competitive and cooperative tasks, revealing distinct neural patterns compared to HCs. These findings provide novel insights into the neurofunctional underpinnings of social cognition and emotion regulation in adolescent depression, with implications for targeted interventions.

### 4.1. Competitive Task Connectivity: Cognitive and Emotional Overload

During competitive maze interactions with parents, adolescents with MDD displayed elevated FC between rIFGoperc and lIFGtriang, as well as lIFGoperc and rIFGtriang. The inferior frontal gyrus complex is central to cognitive control, decision‐making and emotion regulation [32–34]. This hypercoupling may tentatively reflect heightened attentional engagement with social evaluative cues during competition, as depressed youth may allocate excessive cortical resources to process potential negative social feedback. This correlational pattern aligns broadly with the social competition failure hypothesis, which posits exaggerated neural responses to competitive social stress among depressed individuals, potentially correlating with subjective feelings of inferiority and defeat [15, 16].

Increased connectivity between left anterior cingulate gyrus and medial superior frontal gyrus may imply amplified self‐referential processing and mood‐related cortical dysregulation, which could be associated with persistent negative self‐perception in depressed adolescents (Pan, 2022 #182) [35–37]. Enhanced coupling between rSMA and SFGmed/SFGdor may further indicate greater cortical resource expenditure to coordinate behavioral strategies and downregulate unpleasant affect during competitive scenarios [38–40]. Meanwhile, reduced lSFGdor–lPreCG connectivity points to weakened integration between high‐order cognitive planning and motor error monitoring circuits [41–43]. Such altered connectivity patterns may be linked to reduced behavioral flexibility when rapid strategic adjustments are required under time pressure in competitive contexts.

Notably, all above interpretations remain preliminary and speculative. This study did not collect concurrent behavioral metrics including self‐reported emotional reactivity, task reaction time, or competitive performance scores, so we cannot provide direct empirical evidence to verify the proposed cognitive overload or emotional hypersensitivity accounts. Causal relationships between these cortical connectivity shifts and depressive social dysfunction cannot be concluded solely based on fNIRS hemodynamic correlations.

### 4.2. Cooperative Task Connectivity: Social and Emotional Challenges

In cooperative parent–adolescent interaction, the DD group exhibited stronger coupling between lIFGoperc and rORBinf, two cortical regions implicated in emotion processing, social cognition and reward valuation [44, 45]. This elevated connectivity may reflect extra cortical effort dedicated to emotion regulation and interpersonal adaptation during joint cooperation, which could potentially compensate for atypical social reward processing frequently reported in depression [25, 26]. Similarly, strengthened bilateral ACG connectivity suggests amplified self‐monitoring and conflict resolution processing during interpersonal coordination, which may correspond to greater neural burden when maintaining smooth mutual interaction for depressed adolescents [36].

These cortical connectivity shifts partially align with existing theoretical models of socioemotional integration deficits in major depression [13]. Nevertheless, the present cortical‐level fNIRS data alone cannot confirm core impairments in affective resonance or theory‐of‐mind capacity. Standardized behavioral assessments of social cognition are required in future research to validate these correlational neural observations.

### 4.3. Task‐Context Modulated Neural Plasticity

Significant Group × Task interaction effects derived from GLMM analyses robustly demonstrate that atypical prefrontal–cingulate connectivity in adolescent depression is highly context‐dependent, with clearly dissociable cortical signatures emerging under competitive versus cooperative social conditions. Competitive tasks elicited a connectivity profile characterized by hyperconnectivity within emotion‐cognitive control circuits and weakened executive‐motor integration. By contrast, cooperative interaction featured elevated coupling across socioemotional cortical networks, which may represent compensatory cortical recruitment to offset underlying social cognitive limitations [13].

Here we propose a tentative dual‐pathway descriptive framework only to systematically summarize the task‐specific dissociation of frontocingulate connectivity observed in this dataset. This framework functions merely as a descriptive summary of cortical circuit differences and should not be interpreted as a definitive causal neurobiological mechanism explaining social impairment. An important limitation of fNIRS further restricts this interpretation: the imaging modality cannot capture subcortical nuclei, so this descriptive model cannot reflect the full spectrum of brain circuits supporting social cognition [27].

### 4.4. Innovations and Clinical Translational Potential

The current research is among the first to adopt fNIRS hyperscanning to measure real‐time cortical synchrony between depressed adolescents and their primary caregivers during ecologically valid social interaction, delivering higher ecological validity compared to constrained fMRI paradigms [19]. The context‐specific connectivity alterations identified in this work may provide candidate cortical circuits for future neuromodulation research, though these circuits cannot be validated as reliable clinical intervention targets based solely on the present cross‐sectional correlational data.

For illustration, the attenuated lSFGdor–lPreCG connectivity observed exclusively in competitive tasks could serve as a candidate target for follow‐up transcranial magnetic stimulation (TMS) trials to explore whether modulating this circuit improves cognitive flexibility and error monitoring [42]. In parallel, enhanced rSMA–rMFG coupling may be explored as a potential biomarker for fNIRS neurofeedback training targeting behavioral regulation [39]. Clinically, these preliminary correlational findings may offer tentative clues for developing parent–child joint cooperative training programs and cognitive–behavioral interventions targeting negative self‐evaluation triggered by competitive social situations. Large‐scale longitudinal clinical trials combining neural and behavioral assessments are still necessary to verify whether targeted modulation of these cortical circuits produces lasting improvements in real‐world social functioning.

### 4.5. Conclusion and Future Directions

Our results delineate dissociable prefrontal–cingulate cortical connectivity alterations that correlate with social cognitive dysfunction in adolescent major depression, highlighting distinct cortical neural signatures shaped by competitive and cooperative interpersonal contexts. The tentative dual‐pathway descriptive framework proposed in this work may serve as a reference for subsequent research investigating context‐dependent social neural dysfunction, with only preliminary suggestive implications for developing personalized intervention strategies [8].

Several critical limitations must be acknowledged to avoid overinterpretation of the present findings. First, the cross‐sectional study design prohibits any causal inference between abnormal cortical connectivity and depressive social dysfunction; we cannot determine whether atypical frontocingulate coupling is a predisposing vulnerability or a secondary outcome of chronic depressive symptoms [12]. Second, the sample size of depressed and healthy adolescents is relatively limited, and no a priori power analysis was performed to justify the sample size for the large volume of multiple connectivity comparisons. A modest sample may reduce statistical power and limit the generalizability of these connectivity patterns to broader adolescent clinical populations. Third, fNIRS only reliably records hemodynamic signals from superficial cortical gray matter and cannot measure subcortical nuclei such as the amygdala and ventral striatum, restricting comprehensive whole‐brain interpretation of social cognitive impairment mechanisms [27]. Fourth, the study lacks synchronized behavioral indicators of social interaction, including task performance accuracy, subjective emotional ratings, and quantitative interpersonal coordination metrics. As a result, all mechanistic inferences regarding cognitive overload, emotional hypersensitivity and impaired affective resonance remain unvalidated preliminary hypotheses, which cannot be directly supported by the current neural dataset.

Future research should adopt longitudinal designs combined with multimodal imaging techniques (e.g., simultaneous fMRI and fNIRS) paired with standardized social cognitive behavioral batteries to validate and extend these preliminary cortical connectivity observations. Additionally, exploring gene–environment interaction effects may clarify how genetic vulnerability modulates context‐specific frontocingulate connectivity abnormalities in depressed adolescents, further refining precision therapeutic strategies for youth depression.

## Author Contributions

All authors contributed to the conception of the idea, implementing and analyzing the experimental results, writing the manuscript, and read and approved the final manuscript.

## Funding

This work was supported by the National Natural Science Foundation of China (Regional Science Fund Project, Grant 82260878).

## Disclosure

All authors contributed to the article and approved the submitted version.

## Ethics Statement

In this study involving human subjects, ethical standards from the 1964 Declaration of Helsinki were followed. Ethical approval was granted by an Institutional Review Board, ensuring compliance with ethical guidelines and participant rights protection. This study did not involve animal subjects.

## Consent

Informed consent was obtained from the participant for publication, with privacy safeguards.

## Conflicts of Interest

The authors declare no conflicts of interest.

## Data Availability

The raw data supporting the findings of this study are available from the corresponding author upon reasonable request.
